# Advanced Glycation End-Products-, C-Type Lectin- and Cysteinyl/ Leukotriene-Receptors in Distinct Mesenchymal Stromal Cell Populations: Differential Transcriptional Profiles in Response to Inflammation

**DOI:** 10.22074/cellj.2018.5104

**Published:** 2018-03-18

**Authors:** Mehdi Najar, Mohammad Fayyad-Kazan, Gordana Raicevic, Hussein Fayyad-Kazan, Nathalie Meuleman, Dominique Bron, Laurence Lagneaux

**Affiliations:** 1 Laboratory of Clinical Cell Therapy, Institute of Jules Bordet, Brussels, Free University of Brussels (ULB), Brussels, Belgium; 2 Institute of Molecular Biology and Medicine, Free University of Brussels, Gosselies, Belgium; 3 Laboratory of Cancer Biology and Molecular Immunology, Faculty of Sciences I, Lebanese University, Hadath, Lebanon; 4 Experimental Hematology, Institute of Jules Bordet, Free University of Brussels, Waterloo Street, Brussels, Belgium

**Keywords:** Advanced Glycation End-Products Receptor, C-Type Lectin Receptors, Inflammation, Mesenchymal Stromal Cells

## Abstract

**Objective:**

We aimed at characterizing the transcription profiles of immunological receptors associated with the biology of mesenchymal stromal cells (MSCs).

**Materials and Methods:**

In this experimental study, quantitative real time-polymerase chain reaction (qRT- PCR) was performed to establish the transcription profiles of advanced glycation end-products (*RAGE*) receptor, C-type lectin receptors (CLRs, including *DECTIN-1, DECTIN-2* and *MINCLE*), leukotriene B4 (*LTB4*) receptors (*BLT1* and *BLT2*) and cysteinyl leukotrienes (CysLTs) receptors (*CYSLTR1* and *CYSLTR2*) in distinct populations of MSCs grown under basic or inflammatory conditions.

**Results:**

MSCs derived from adipose tissue (AT), foreskin (FSK), Wharton’s jelly (WJ) and bone marrow (BM) exhibited significantly different transcription levels for these genes. Interestingly, these transcription profiles substantially changed following exposure of MSCs to inflammatory signals.

**Conclusion:**

Collectively, for the first time, our data highlights that MSCs depending on their tissue-source, present several relevant receptors potentially involved in the regulation of inflammatory and immunological responses. Understanding the roles of these receptors within MSCs immunobiology will incontestably improve the efficiency of utilization of MSCs during cell-based therapies.

## Introduction

Mesenchymal stromal cells (MSCs) are multipotent progenitor cells characterized by fibroblast-like shape, high self-renewal capacity and multilineage differentiation potential. Initially isolated from the bone marrow (BM), MSCs have been successfully derived from several other tissues including adipose tissue (AT), Wharton’sjelly (WJ) of the umbilical cord and foreskin (FSK) (1, 2). Besides their tissue regenerative capacities, MSCs display important immunomodulatory functions allowing their use as immunotherapeutics (3). 

This is of immense importance that while MSCs are not immunogenic, they have the ability to modulate innate and adaptive immune responses by a network of regulatory pathways that converge and compete to establish a tolerogenic state (4). Following administration, MSCs can migrate to injured sites thus promoting tissue repair. The ability to communicate with the surrounding environment is a major requirement for the therapeutic process initiated by MSCs. However, studies investigating *in vivo* effects of MSCs have yielded conflicting results (5, 6). Thus, determining immunobiological criteria of a given immunotherapeutic intervention is highly important for achieving an efficient approach (7).

Interestingly, *in vivo* responses of transplanted MSCs could be triggered by various danger signals, such as inflammation, infection, damage and hypoxia (8). So, the environment can greatly influence the behavior of MSCs since they can actively sense different signals and accordingly, modulate their biological functions (9). Since inflammation substantially modulates the properties of MSCs during infection, it is considered an important regulator of cell biology (10, 11). However, the response of MSCs to other critical danger signals such as leukotrienes and/or cysteinyl leukotrienes, advanced glycation end-products (AGEs), pathogen-associated molecular patterns (PAMPs) and damage-associated molecular patterns (DAMPs) depends on the expression of their related receptors. The expression patterns of such receptors within MSCs are still poorly characterized.

In this work, for the first time, we have demonstrated that MSCs, according to their tissue-origins, express distinct receptors (RAGE, CLRs, BLT1, BLT2, CYSLTR1 and CYSLTR2) which are associated with danger signals. Furthermore, inflammation greatly influences the transcription patterns of these receptors. Understanding the roles of these receptors, being considered as inducers, sensors, and mediators of the inflammatory processes (12, 13), within MSCs’ immunobiology will incontestably improve the efficiency of employment of MSCs in cell- based therapies.

## Materials and Methods

### Ethical guidelines

The present experimental study was conducted in accordance with the Declaration of Helsinki (1964) and was approved by the local Ethics Committee of the “Institut Jules Bordet” (Belgium). All samples were obtained from healthy donors who gave informed written consent before initiation of the study.

### Isolation and culture of human mesenchymal stromal cells

AT-MSCs, BM-MSCs, FSK-MSCs and WJ-MSCs were isolated from seven independent healthy donors as previously described (2, 14). Briefly, cell cultures were incubated at 37°C in a humidified atmosphere with 5% CO_2_. After 48 hours, non-adherent cells were removed upon changing the medium. When sub-confluency (80-90%) was achieved, adherent cells were harvested by TrypLE Select solution (Lonza Belgium) and then expanded at a lower density (1,000 cells/c). In order to assess the impact of an inflammatory environment, cells were cultivated under both basic and inflammatory conditions as previously described (15). Briefly, cells were treated (overnight) with a cocktail of pro-inflammatory cytokines containing interleukin 1-beta (IL-1ß, Peprotech, Rocky Hill, NJ, USA, 25 ng/ml), tumor necrosis factor-alpha (TNF-α, 50 ng/ml), *interferon-alpha* (IFN-α, 3000 U/ml or 10 ng/ml) and IFN-γ (1000 U/ml or 50 ng/ml) (all purchased from Prospec Inc., Rehovot, Israel).

### Quantitative real-time polymerase chain reaction

Total RNA was extracted using TriPure Isolation Reagent according to the manufacturer’s guidelines (Roche Applied Science, Vilvoorde, Belgium). Reverse transcription reaction was applied for 1 mg RNA using qScript cDNA SuperMix (Quanta Biosciences, USA). Next, mRNA levels were quantified by real-time PCR using 20 ng of cDNA, SYBR Green PCR Master Mix (Applied Biosystems, Lennik, Belgium) and 0.32 mM forward and reverse primers. *GAPDH* was used as a housekeeping gene. ABI Prism 7900 HT system (Applied Biosystems, USA) was used to perform the amplification reactions. In all cases, dissociation curves were generated and the specificity of the PCR reactions was checked. The comparative ΔΔCt method was followed for data analysis. All qPCR reactions were performed in triplicates. To evaluate the fold change, data were normalized against the *GAPDH* genes to obtain the ΔCt and calibrated using the geometric mean of the *GAPDH* ΔCt to generate the ΔΔCt. Fold changes were then calculated as fold change=2^-ΔΔCt^. The sequences of the used primers are indicated in Table 1. 

**Table 1:** Quantitative real time-polymerase chain reaction (qRT-PCR) primers used in this study


Transcripts	Primer sequencing (5ˊ-3ˊ)

*GAPDH*	F: AATCCCATCACCATCTTCCA
	R: TGGACTCCACGACGTACTCA
*DECTIN-1*	F: AAAGGATCGTGTGCTGCATCT
	R: TACCAAGCATAGGATTCCCAAAAT
*DECTIN-2*	F: CATTCAAGTCTCACCTGCTTCAGT
	R: TCCAAGAAGCTGGGCAACAT
*MINCLE*	F: ACCAGGTTGTCGAGGGTCCAGT
	R: CCCAGAAGCTCAGAGACTTTGTC
*RAGE*	F: TGGAACCGTAACCCTGACCT
	R: CGATGATGCTGATGCTGACA
*BLT1*	F: CCTGAAAAGGTGCAGAAGC
	R: AAAAAGGGAGCAGTGAGCAA
*BLT2*	F: CTTCTCATCGGGCATCACAG
	R: ATCCTTCTGGGCCTACAGGT
*CYSLTR1*	F: TCCTTAGAATGCAGAAGTCCGTG
	R:AAATATAGGAGAGGGTCAAAGCAA
*CYSLTR2*	F: GCTGATCATTCGGGTTCTGT
	R: GGTGATGATGATGGTGGTCA


### Statistical analysis 

Presented data correspond to means ± SEM of three independent experiments and statistically significant differences in gene expression between control and treated cells were determined using unpaired Mann-Whitney U test. P<0.05 were considered significant.

## Results

Unravelling the receptors, particularly those related to cell danger/injury, that are expressed by MSCs will ultimately determine MSCs immunotherapeutic profile and function and consequently enhance their therapeutic value. Accordingly, using quantitative real-time PCR (qRT-PCR) we examined the transcription profiles of several relevant receptors (<italic>BLT1, BLT2, CYSLTR1, CYSLTR2, RAGE, DECTIN-1, DECTIN-2</italic> and <italic>MINCLE</italic>) (Table 2) in different types of MSCs (BM, WJ, AT and FSK) cultivated under basic (non-inflammatory) or inflammatory conditions. Globally, the transcription pattern of these receptors varied according to MSCs tissue-origin and was greatly influenced by inflammation.

**Table 2:** List of receptors-encoding genes included in this study


Receptors for:	Gene

Advanced glycation end-products (AGE)	
Receptor for advanced glycation end-products (RAGE)	AGER
Leukotriene B4 (LTB4)	
Leukotriene B4 receptor 1 (BLT1)	LTB4R
Leukotriene B4 receptor 2 (BLT2)	LTB4R2
Cysteinyl leukotrienes (CysLTs)	
Cysteinyl leukotriene receptor 1 (CYSLTR1)	CysLTR1
Cysteinyl leukotriene receptor 2 (CYSLTR2)	CysLTR2
C-type lectin	
C-type lectin domain family 7 member A (CLEC7A; DECTIN-1)	CLEC7A
C-type lectin domain family 6, member A (CLEC6A; DECTIN-2)	CLEC6A
C-Type Lectin Domain Family 4 Member E (CLEC4E; MINCLE)	CLEC4E


## Compliance of mesenchymal stromal cells with International Society for Cellular Therapy criteria

**Fig.1:**
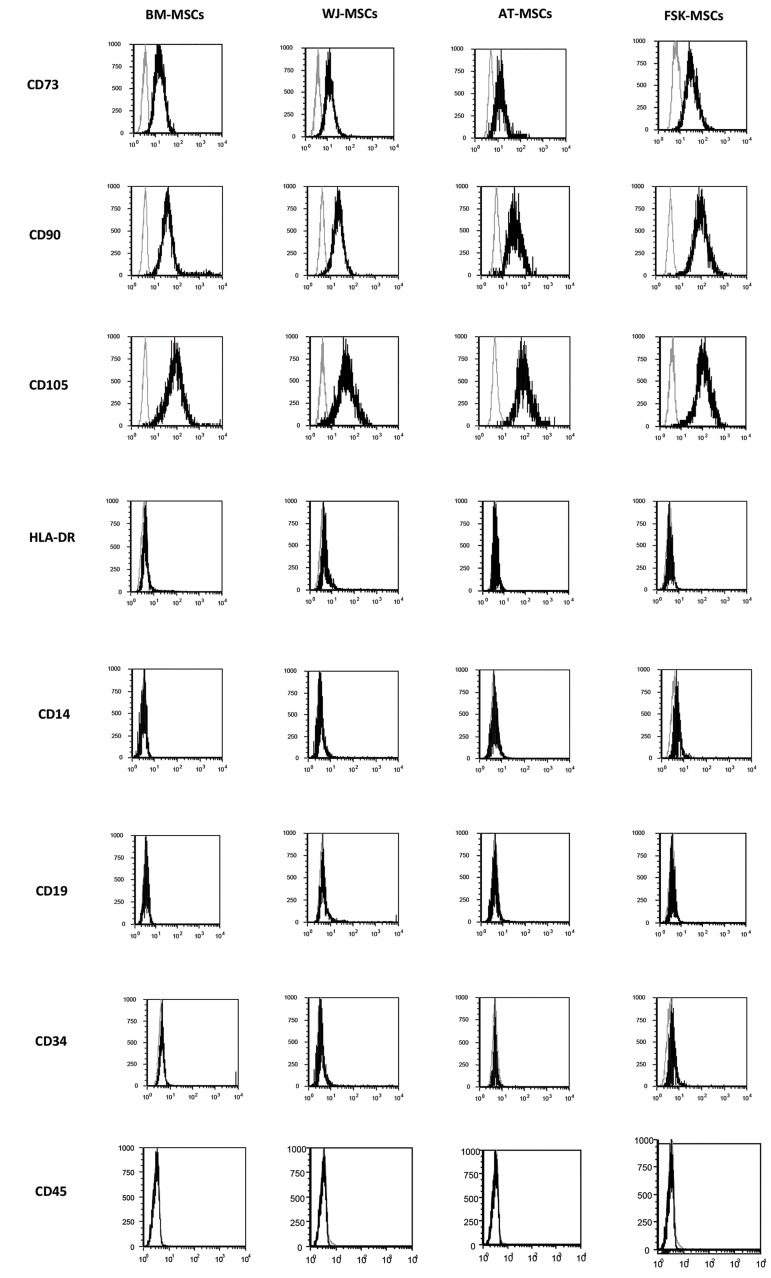
Flow cytometry analysis was used to establish the phenotype of mesenchymal stromal cells (MSCs) derived from distinct tissues according to ISCT criteria. A panel of fluorochrome-labelled monoclonal antibodies was used to assess the expression patterns of different surface markers (Gray line: isotype fluorescence; black line: antibody-specific fluorescence).

**Fig.2:**
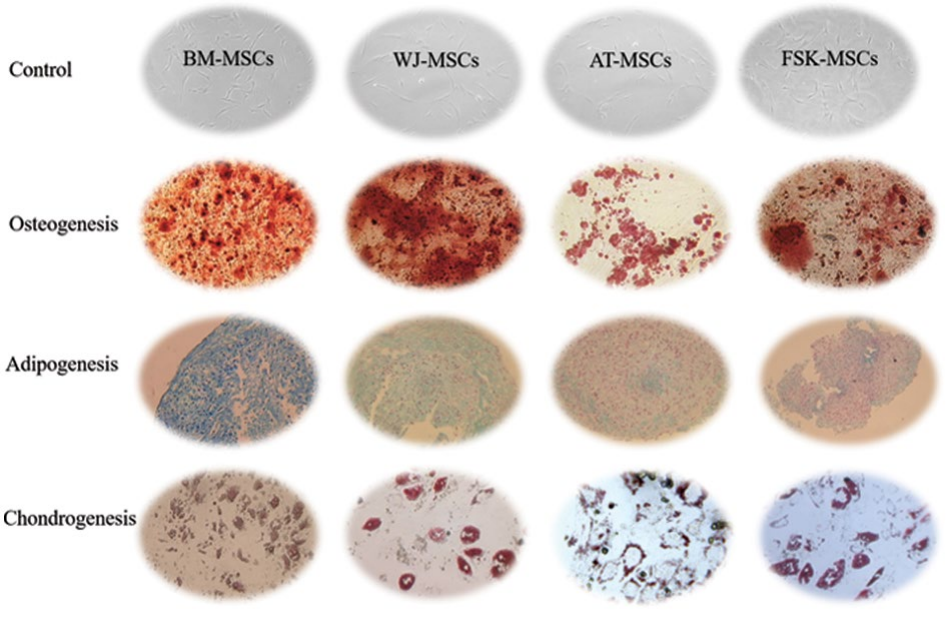
Representative images demonstrating the multilineage differentiation potential of mesenchymal stromal cells (MSCs) derived from distinct C tissues as assessed upon using both specific lineage induction medium and staining techniques. For adipocytes, lipid vacuoles were stained byOil Red O. For osteoblasts, calcium deposit was stained by Alizarin red. For chondrogenic pellets, proteoglycans synthesis was stained by Alician blue.

Cell-based therapy requires a well-defined and identified cellular product. Thus, before any experimental assay, the studied cells were critically characterized according to with International Society for Cellular Therapy (ISCT) criteria. We confirmed that different MSCs used in this study, were compliant with ISCT criteria (16). Indeed, they presented a fibroblastic morphology and a high capacity to adhere to plastic. Flow cytometry analysis demonstrated that distinct MSCs were positive (>95%) for CD73, CD90 and CD105 but negative (<5%) for CD14, CD19, CD34, CD45 and HLA-DR (Fig .1). Moreover, these MSCs exhibited a multilineage potential, as they were able to generate adipocytes, osteoblasts and chondrocytes (Fig .2).

## Expression and modulation of *BLT1* and *BLT2*

**Fig.3:**
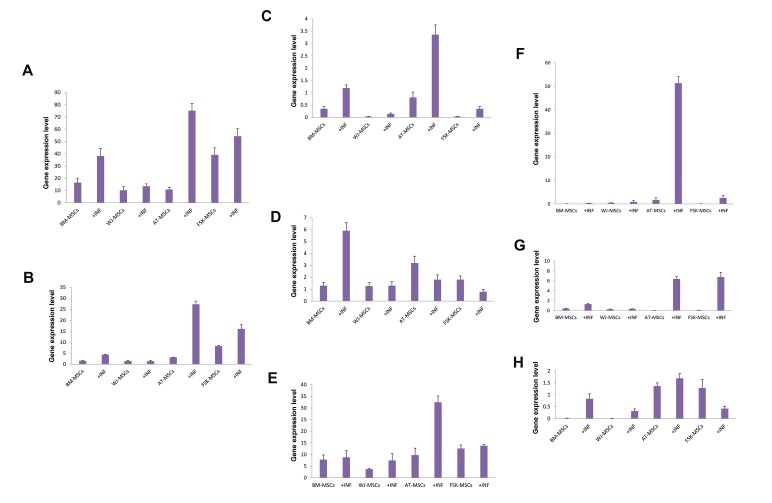
Characterization of *BLT1, BLT2, CYSLTR1, CYSLTR2, RAGE, DECTIN-1, DECTIN-2* and *MINCLE* transcription profile in mesenchymal stromal cells (MSCs) of different origins under basic or inflammatory conditions (+INF). Total RNA was isolated from BM-, WJ-, AT- and FSK-MSCs being cultivated in the absence (basic condition) or presence of inflammatory cocktail. GAPDH-normalized A. *BLT1, B. BLT2, C. CYSLTR1, D. CYSLTR2, E. RAGE, F. DECTIN-1, G. DECTIN-2,* and H. *MINCLE* mRNA levels were assessed using quantitative real time-polymerase chain reaction (qRT-PCR). Reported values represent the averages of three independent experiments ± SEM. The statistical significance was determined using Mann-Whitney U- test.

Under basic growth conditions, *BLT1* and *BLT2* genes were constitutively transcribed in all MSCs, but differences in the mRNA levels were noted. The highest transcription levels were observed in FSK-MSCs. Following inflammation-priming, *BLT1* and *BLT2* transcription was strongly induced in AT-MSCs (7-fold increase in case of <italic>BLT1</italic> and 9-fold increase in case of <italic>BLT2</italic>), moderately elevated in BM- and FSK-MSCs (2- and 1.5-fold increase, respectively in case of <italic>BLT1</italic>; 2.5- and 2-fold increase, respectively in case of *BLT2*) but showed no change in WJ-MSCs (Fig .3A, B).

## Expression and modulation of *CYSLTR1* and *CYSLTR2*

Under basic growth conditions, *CYSLTR1* gene appeared to be transcribed in BM-MSCs and AT-MSCs with the latter showing higher *CYSLTR1* mRNA levels. However, a very low, if any, transcription was detected in WJ- and FSK-MSCs (Fig .3C). On the other hand, *CYSLTR2* gene was transcribed in different MSCs studied, with AT-MSCs showing the highest transcription levels, followed by FSK-MSCs, BM-MSCs and finally WJ-MSCs (Fig .3D). Interestingly, inflammation priming significantly enhanced *CYSLTR1* transcription levels in AT-MSCs and BM-MSCs (4-fold increase in both cases) (Fig .3C). In case of *CYSLTR2*, and following inflammation priming, the mRNA levels were specifically and significantly increased in BM-MSCs (5-fold increase). Under inflammatory conditions, WJ-MSCs and FSK- MSCs did not show significant alterations in *CYSLTR1* and *CYSLTR2* transcription levels (Fig .3D).

## Expression and modulation of RAGE

Under basic growth conditions, the transcription profile of *RAGE* was comparable among different MSCs. The highest levels of RAGE mRNA were exhibited by FSK-MSCs followed by AT- and BM-MSCs whereas WJ-MSCs displayed less RAGE mRNA than the others. Intriguingly, inflammation priming specifically and strikingly enhanced *RAGE* transcription in AT-MSCs (3.5-fold increase), while no significant alterations were observed in the other cell types (Fig .3E). 

## Expression and modulation of CLRs

Under basic growth conditions, all types of MSCs showed minimal, if any, transcription of *CLEC7A (DECTIN-1)* and following inflammation-priming, only AT-MSCs demonstrated a huge induction of DECTIN-1 mRNA levels (30-fold increase). To a lesser extent, BM-MSCs showed a slight but significant DECTIN-1 induction (5-fold increase) (Fig .3F). In parallel, *CLEC6A (DECTIN-2)* was also minimally transcribed in different types of MSCs under basic growth conditions. However, inflammation priming induced a substantial increase in DECTIN-2 mRNA levels in AT- and FSK-MSCs (126- and 84-fold increase, respectively). DECTIN-2 mRNA was moderately increased in BM-MSCs (3-fold increase) but remained unchanged in WJ-MSCs (Fig .3G). Under basic growth conditions, *CLEC4E* gene *(Mincle)* appeared to be minimally transcribed in both AT- and FSK-MSCs but neither in BM- nor WJ-MSCs. Inflammation priming had no significant impact on the transcription of Mincle in different types of MSCs (Fig .3H). 

## Discussion

While MSCs are non-immunogenic, they exert marked immunomodulatory activities, thus, they have emerged as a promising immunotherapeutic tool (4, 17). In vivo, following their migration to the local injured tissue, the responses of infused MSCs are influenced by different danger signals such as infection, inflammation and hypoxia (9). Importantly, MSCs are environmentally responsive cells that actively sense their surroundings and modulate their biological functions accordingly. As important mediators of danger/injury signals (12, 15), we determined and compared the transcription profiles of *RAGE, CLRs, BLT1, BLT2, CYSLTR1* and *CYSLTR2* in different types of MSCs (BM, WJ, AT, and FSK) and investigated the impact of inflammation on them. 

In such context, inflammation is known to critically modulate the properties of stem cells (10). Moreover, differences in the immunologic profiles and immunomodulatory activities displayed by MSCs, derived from different tissue-sources, have been reported (18, 19). We think that efficient use of MSCs requires such characterization and comprehension of the receptors associated with cell danger/injury, which could ultimately determine the therapeutic function of MSCs. After confirming that the cells used in this study are compliant with ISCT criteria, for the first time, we evidenced a differential transcription pattern of these receptors with regard to both MSC-tissue origin and surrounding inflammatory status.

Leukotrienes and/or cysteinyl leukotrienes are important regulators of immune and inflammatory responses which exert their effects by binding to their respective receptors. Leukotriene B4 *(LTB4)* is a highly potent inflammatory mediator acting through two G protein-coupled seven-transmembrane domain receptors (GPCR) known as *BLT1* and *BLT2* (20). In addition to their different affinities and specificities for LTB, these two receptors show distinct expressions with *BLT1* being mainly expressed in leukocytes, whilst *BLT2* showing ubiquitous expression. On the other hand, the cysteinyl leukotrienes (Cys-LTs) *(LTC4, LTD4, LTE4</italic> and <italic>LTF4)* are a family of potent bioactive lipids that have amino acid cysteine in their structure. They act upon binding to target cell-surface GPCRs, *CYSLTR1* and *CYSLTR2* (21).

By transducing the signals of LTB4 and cys-LTs, these receptors are involved in the recruitment and activation of leukocytes as well as the stimulation of the immune response (22). In this report, we showed that *BLT1* and *BLT2* are differentially transcribed in the different types of MSCs, and inflammatory signals can further enhance their expression levels in AT-, BM- and FSK-MSCs. In fact, very few information is known about the role of LTB4 receptors (i.e. *BLT1* and *BLT2*) in regulating MSCs’ biology. A previous report indicated that *BLT1* and *BLT2* play opposite roles during regulation of umbilical cord- derived MSCs proliferation (23). 

Regarding the transcription profile of *CYSLTR1* and *CYSLTR2, CYSLTR1* was mainly transcribed in BM- and AT-MSCs whilst *CYSLTR2* was transcribed in all MSC types. Moreover, inflammation-priming up-regulated, though to different extents, the mRNA levels of both receptors in a tissue-origin dependent manner. Remarkably, literature mining did not identify any previously described role for *CYSLTR1* and *CYSLTR2* in regulating MSCs’ behavior. MSCs, by presenting *BLT1* and *BLT2* as well as *CYSLTR1* and *CYSLTR2* and most importantly by adequately adjusting their expression as observed in an inflammatory setting, may contribute to the inflammatory process. Indeed, MSCs have been shown to significantly target lymphocyte extravasation and trafficking as a part of their immunomodulatory effects (24).

Through their corresponding receptors, LTB4 and CysLTs are partially responsible for higher IL-10 but lower IL-12/p40 production by dendritic cells (DCs) but have no effect on IL-6 release. By modulating the secretion profile of DCs, LTB4 as well as CysLTs may initiate T helper 2-type immune responses (21, 25). A parallel between DC and MSCs could be thus supposed. By expressing these receptors, MSCs may change their cytokine secretion profile in response to the presence of leukotrienes. However, MSCs do not express IL-10 by themselves but modulate the lymphocyte IL-10/CD210 axis (26); however, they are able to secrete substantial amounts of IL-6 (27). MSCs, by altering the cytokine secretion profile of immune cells, induce a Th2-polarized immune response that leads to T-cell inhibition (28, 29).

Advanced glycosylation end products (AGEs) correspond to modified protein or lipid structures resulting from the non-enzymatic glycation and oxidization reactions following contact with reducing sugars and may generate an inflammatory response (30). Due to their accumulation in various cell types, the extracellular and/or intracellular structure and function of AGEs may vary following engagement with RAGE (Advanced glycosylation end product-specific receptor) (31). RAGE is a member of the immunoglobulin superfamily of cell surface molecules and is expressed by a variety of cells. Binding of ligands to RAGE triggers different signal transduction mechanisms that are mainly involved in inflammation (32).

Due to its inflammatory role in innate immunity and ability to recognize a common structural motif exhibited by a set of ligands, RAGE is also considered as a pattern recognition receptor (PRR). Indeed, RAGE may mediate inflammasome activation and subsequent release of pro-inflammatory mediators, thus contributing to the propagation of the innate immune response (33). 

In this study, we observed that the transcription profile of *RAGE* varied considerably among different MSCs tested. In particular, AT-MSCs showed constitutively high amount of *RAGE* mRNA which subsequently increased following inflammation-priming. In the literature, two animal models have demonstrated a possible role for *RAGE* in the immunomodulatory potential of MSCs. In fact, it has been shown that BM-MSCs overexpressing *RAGE* could competitively bind the high mobility group box chromosomal protein 1 (HMGB1), an important pro- inflammatory molecule, thus reducing the subsequent immuno-inflammatory response in a rat model of acute liver failure (ALF) (34). 

Another study, performed in a mouse model of rheumatoid arthritis (RA) demonstrated that overexpression of *RAGE* in AT-MSCs optimized their immunoregulatory properties by decreasing their production of pro-inflammatory mediators (such as IL-1ß and IL-6) and increasing the expression of regulatory molecules (such as IL-10 and TGF-ß). These immunological changes inhibited the differentiation of Th1 as well as Th17 cells, and reciprocally induced T regulatory cell expansion (35). As we observed that inflammation induced high RAGE transcription within AT-MSCs, we can hypothesize that this effect will promote AT-MSCs immunomodulatory functions and consequently enhance their therapeutic effects. 

CLRs constitute a large family of soluble transmembrane proteins that possess one or more C-type lectin-like domains (CTLD). Different members of this family including DECTIN-1, DECTIN-2 and MINCLE, are considered to be PRRs. CLRs are primarily expressed by immune cells but might be found on other cell types. By sensing molecules that are associated with infection (i.e. PAMPs) or tissue damage (i.e. DAMPs), CLRs contribute to the regulation of the inflammatory and immune response (36). 

In the present study, we reported that the transcription profile of CLRs within MSCs is closely dependant on their tissue-origins as constitutive differences in the expression levels of *DECTIN-1, DECTIN-2,* and *MINCLE* were noted among different types of MSCs. Moreover, inflammation specifically increased the expression of *DECTIN-1* and *DECTIN-2* while no evident impact could be concluded for MINCLE. Importantly, the inflammation-induced increase was not general, as *DECTIN-1* was enhanced particularly in AT-MSCs and *DECTIN-2* was restricted in BM-, AT- and FSK-MSCs. CLRs can activate distinct signalling cascades that trigger the production of certain cytokines which specify the fates of T-cell polarization. Signalling pathways activated by CLRs either directly regulate the production of pro-inflammatory cytokines through MAPK (Mitogen-activated protein kinase) and NFkB (nuclear factor kappa-light-chain-enhancer of activated B cells) activation or indirectly by modulating Toll-like receptor (TLR)-mediated immune complexes (37).

Interestingly, MSCs display different TLR expression profiles and inflammation was shown to differentially alter these expression patterns in a manner dependent on MSCs tissue source (38). Ligand-mediated activation of these receptors can induce several immune activities including DC maturation, production of reactive oxygen species (ROS), secretion of pro-inflammatory cytokines and development of Th1, Th17 and CD8 cytotoxic lymphocytes (CTLs). The role of DECTIN-1, DECTIN-2 and MINCLE in orchestrating MSCs function is still poorly characterized. A recent study suggested a role for DECTIN-2 in increasing the osteogenic activity and cartilage repair potential of human BM-MSCs (39). 

According to our observations, a role for each of these receptors in modulating MSCs biology, particularly during inflammation, could be thus speculated. As a result, induced signaling pathways of either of the studied receptors within MSCs, can precisely control the expression or modulation of several cytokines that govern the inflammatory and immune response.

## Conclusion

Before translating MSCs-based therapy into clinical practice, the environment of the host at the moment of transplantation should be well defined and mimicked *in vitro*. MSCs actively sense their surroundings and accordingly modulate their biological functions. The capacity of MSCs to sense new danger/injury signals such as leukotrienes and/or cysteinyl leukotrienes, AGEs, PAMPs and DAMPs have to be taken into account by characterizing and identifying their receptors. Our results revealed differential transcription profiles of these receptors in a manner dependent on both the MSCs tissue source and the inflammatory status of the surroundings. The environment is an essential parameter triggering the MSCs to adapt either anti-inflammatory or pro- inflammatory phenotype and consequently, determining their therapeutic efficacy. Since these receptors are important for the inflammatory and immune response, further investigation of the signaling pathways and the biological effects that they promote is required to achieve the optimal therapeutic effect of MSCs. 
